# SOCIOECONOMIC DETERMINANTS OF RACIAL INEQUALITIES IN COGNITIVE FUNCTIONING AMONG ADULTS 50+ IN THE US AND BRAZIL

**DOI:** 10.1093/geroni/igae098.1063

**Published:** 2024-12-31

**Authors:** Mateo Farina

**Affiliations:** University of Texas at Austin, Austin, Texas, United States

## Abstract

Racial inequalities in cognitive health are well-established. Black older adults have worse cognitive health than White older adults. However, evidence has been primarily found using data on the U.S. population, limiting scientific insight into how historically and socially constructed racial structures impact racial inequalities in cognitive health. Using nationally, representative studies from the United States (HRS) and Brazil (ELSI-Brazil), I examine racial inequalities in cognitive functioning (global and domain-specific) and their life-course socioeconomic determinants. I use a series of linear regression models to examine racial differences in cognitive functioning and to determine how accounting for life course socioeconomic conditions (retrospective childhood socioeconomic status, educational attainment, and adulthood income adjusted for household size) attenuate observed differences. Results show different patterns in racial inequalities in cognitive functioning across countries, with Black Americans having the lowest cognitive functioning in the United States and Asian Brazilians having the lowest cognitive functioning in Brazil. In Brazil and the United States, White older adults had the highest levels of cognitive functioning. However, the magnitude of racial inequalities differed by country, with preliminary findings showing greater differences in the United States than in Brazil. Lastly, regardless of country, childhood socioeconomic conditions and educational attainment greatly attenuated racial differences in cognitive functioning, reducing some racial inequalities to non-significance, indicating that the early life opportunities in establishing cognitive functioning may be substantially contributing to racial inequalities in both contexts. This research points to the need to consider context in understanding how and why racial inequalities in cognitive health arise.

